# Trends and forecasts of the prevalence and mortality of Alzheimer’s disease and other dementias in China

**DOI:** 10.3389/fpubh.2025.1616232

**Published:** 2025-07-22

**Authors:** Mingxuan Hou, Ling Yang

**Affiliations:** ^1^Department of Geriatrics, Shanghai Fourth People's Hospital Affiliated to Tongji University, Shanghai, China; ^2^School of Exercise and Health, Shanghai University of Sport, Shanghai, China

**Keywords:** Alzheimer’s disease, dementia, prevalence, mortality, prediction, China

## Abstract

**Background:**

As one of the most aging populations in the world, China has experienced a continuous increase in the disease burden of Alzheimer’s Disease (AD) and other dementia (ADRD). This study aims to analyze the trends in prevalence and mortality rates of AD and related dementia in China from 1990 to 2021 and to predict the disease burden by 2040.

**Methods:**

Data was extracted from the Global Burden of Disease Study 2021 (GBD 2021). Joinpoint regression was used to identify significant changes in trends, while Age–Period–Cohort (APC) models were applied to disentangle age, period, and cohort effects. Bayesian Age–Period–Cohort (BAPC) modeling was used for future projections. In addition, we conducted a risk factor analysis of AD-related mortality attributable to smoking, high body mass index (BMI), and high fasting plasma glucose (FPG).

**Results:**

In 2021, the age-standardized prevalence of AD and other dementia in China was 1,194 per 100,000 (95% CI: 1018–1,383), and the mortality rate was 35 per 100,000 (95% CI: 9–93). The disease burden was significantly higher in females compared to males (prevalence: females 1,559 per 100,000 vs. males 846 per 100,000). Joinpoint analysis showed a significant increase in both prevalence and mortality rates after 2019 (male APC = 2.81%, female APC = 3.76%). Risk factor analysis highlighted obesity (AAPC = 9.87%) as the leading contributor to AD mortality in China, surpassing global averages. The BAPC model predicted that by 2040, the prevalence in females would increase to 1,180 per 100,000, while the mortality rate for males would stabilize at 17.6 per 100,000. Our results indicate a substantial increase in disease burden, underscoring the urgent need for strategic interventions and resource allocation to mitigate the future impact of dementia-related conditions in China.

**Conclusion:**

The disease burden of AD and related dementia in China continues to rise, necessitating enhanced early screening, optimized healthcare resource allocation, and targeted intervention strategies for high-risk female populations.

## Background

Dementia is a clinical syndrome characterized by progressive decline in two or more cognitive domains, including memory, language, executive and visuospatial functions, personality, and behavior, leading to the loss of the ability to perform instrumental and/or basic activities of daily living. Alzheimer’s disease (AD) is characterized by distinct patterns of age-related cognitive and functional decline coupled with specific neuropathological changes (1).

In 2021 roughly 57 million people worldwide lived with dementia, and AD is the most common cause of dementia, accounting for 80% of all dementia diagnoses (2). Numerous studies project that global dementia prevalence will nearly double every two decades. For example, one synthesis of global data reported 43.8 million dementia cases in 2016 (up 117% since 1990) and forecast 152 million by 2050 (3). These trends reflect aging populations and highlight that dementia imposes heavy burdens on individuals, families, and health systems worldwide. No cure currently exists, so prevention by addressing modifiable risk factors has become a focus (3).

As the world’s most populous country, China has experienced rapid population aging, leading to an increase in the prevalence of age-related diseases such as AD. Consequently, China now has the largest older adult population in the world, and the largest number of AD patients, increasing the aging burden and the healthcare resource burden for AD treatment (4, 5). However, research on the disease burden of AD and other dementia in China remains limited. Many studies rely on extrapolations or select cohorts, and systematic data on national trends in AD prevalence and mortality have been limited. Existing analyses using global burden models suggest China’s AD burden is extremely high – for example, Liu et al. reported that by 2021 China’s AD prevalence and incidence burden had grown threefold since 1990 (6) – but there is only a few reviews specifically focused on China. Moreover, most of the therapies we used now have just begun to put it into clinical use (7).

Given the rapid changes in demographics and health profiles, there is an urgent need for credible data on China’s AD and dementia trends. The existing literature lacks comprehensive analysis of long-term trends in prevalence, incidence, and mortality of AD and other dementias (ADRD) in China, as well as an assessment of evolving risk factors. Many studies report isolated statistics such as one-time surveys or mortality rates in specific regions, and often do not contextualize them within broader patterns.

This study uses data from the GBD 2021 to analyze the trends in the prevalence and mortality of AD and other dementia in China from 1990 to 2021 and predicts future trends up to 2040.

## Materials and methods

### Study design

This study adopted a retrospective observational design to analyze the trends in the prevalence and mortality of Alzheimer’s Disease (AD) and other dementia (ADRD) in China from 1990 to 2021 and predicts future trends up to 2040. We obtained data on Alzheimer’s disease and other dementias for China from the Global Burden of Disease (GBD) 2021 database. The GBD database integrates data from vital registration systems, health surveys, hospital records, and disease registries, using Bayesian meta-regression models to adjust for missing or sparse data and to ensure cross-country comparability (8).

### Data source

Data was obtained from the GBD 2021 database, which includes detailed health data from 195 countries and regions globally from 1990 to the present, covering over 300 diseases and injuries, and more than 70 risk factors (9). We downloaded data on the prevalence, incidence, mortality, and DALYs of AD and other dementia in China, and used age-standardized rates to reflect the disease burden trend. The insidious onset of the disease may compromise the accuracy of dementia incidence measurements. Therefore, we selected prevalence and mortality rates for detailed analysis and projected their future trends (10). The estimated population of China was taken from the United Nations World Population Prospects 2024 Revision: World Population Prospects

### Data extraction

Dementia is a progressive, degenerative, and chronic neurological disorder typified by cognitive dysfunctions that interfere with daily functioning. In this study, Alzheimer’s disease and other dementia were classified according to the *Diagnostic and Statistical Manual of Mental Disorders* (DSM) III, IV, or V, or the Ninth and Tenth Revisions of the *International Classification of Diseases* ICD case definitions as the reference (9). From the GBD Results Tool (GBD visualizations via IHME), we extracted yearly numbers and rates for dementia prevalence and mortality in China from 1990 through 2021, stratified by sex and 5-year age group. We restricted analysis to adults aged ≥40, since dementia is rare at younger ages. We also obtained age-standardized prevalence rates (ASPR) and age-standardized mortality rates (ASMR) per 100,000, which GBD calculates using the World Health Organization’s standard population weights. These standardized rates allow comparison over time and between sexes (11). The GBD data stream is already fully modeled and typically contains no missing values.

### Statistical analysis

#### Joinpoint regression analysis

We used Joinpoint Regression software to identify changes in trends of the age-standardized rates (ASPR and ASMR) and trends related to risk factors over time. Joinpoint regression analysis was conducted using version 5.3.0.0 of Joinpoint software. This statistical method helps identify significant points where trends change. The model uses the least squares method to estimate trends in disease rates, avoiding the non-objectivity of typical trend analysis based on linear trends. We analyzed the annual percentage change (APC), the average annual percentage change (AAPC), and their 95% confidence intervals (CI). A statistical significance was considered when *p* < 0.05 (12).

#### Age-period-cohort analysis (APC model)

To evaluate the separate contributions of age, period, and birth cohort, we fitted log-linear APC models to the age-specific rates. The APC model is commonly used in sociology and epidemiology. Based on the Poisson distribution, the APC model reflects the time trend of incidence or mortality by age, period, and cohort (12). The general expression is given by

log(E)=αage1+βperiod1+γcohort+μ+log(θ)

where E represents the expected rate; α , β and γ denote the effects of age, period, and cohort, respectively, and log(θ) is the offset effect (13, 14). However, due to the linear dependency among age, period, and cohort, estimating a unique set of effects for each age, period, and cohort remains challenging, leading to potential identifiability issues (12). To mitigate this, the study adopted a grouping approach by dividing the years 1990 to 2021 into five-year intervals. Additionally, since the Global Burden of Disease (GBD) already categorizes age groups in five-year increments, the categories were redefined for the APC (Age-Period-Cohort) analysis to reduce the impact of collinearity (15).

#### Bayesian age-period-cohort (BAPC) model

For projections beyond 2021, we applied Bayesian APC models using Integrated Nested Laplace Approximation (INLA) in R 4.4.2 (16). The BAPC model combines the APC model with Bayesian methods, integrating sample information with prior knowledge to yield predicted results. This model was implemented using the INLA package in R 4.4.2 software (17). To meet the requirements of the BAPC prediction model, we included the 40–44 age group and consolidated individuals aged 95 and above into a single group.

## Results

### Descriptive statistics

In 2021, China had 16,990,827 new cases (95% CI: 14,488,494–19,672,741) and 491,774 deaths (95% CI: 4,947,154–22,219,154) due to Alzheimer’s Disease and Related Dementia (ADRD). In 2019, the age-standardized incidence, prevalence, DALYs, and mortality rates for ADRD were 205 per 100,000 (95% CI: 176–236), 1,194 per 100,000 (95% CI: 1018–1,383), 708 per 100,000 (95% CI: 348–1,562), and 35 per 100,000 (95% CI: 9–93), respectively. Table 1 shows the total cases and age-standardized rates for both males and females. It is evident that the disease burden of ADRD is higher in females than in males.

**Table 1:** All-age cases and age-standardized incidence, prevalence, DALYs, and mortality of ADRD in China, 2021.

Metric	All-age numbers (95% CI)			Age-standardized rates (95% CI) per 100,000		
	Total	Male	Female	Total	Male	Female
Incidence	2,914,112 (2504728–3,350,743)	1,077,297 (908448–1,248,194)	1,836,815 (1593651–2,101,343)	205 (176–236)	148 (125–171)	264 (229–303)
Prevalence	16,990,827 (14488494–19,672,741)	6,162,198 (5142286–7,141,800)	10,828,630 (9315735–12,515,957)	1,194 (1018–1,383)	846 (706–981)	1,559 (1341–1802)
DALYs	10,072,478 (4947154–22,219,154)	3,572,279 (1694716–8,148,478)	6,500,199 (3171765–13,681,029)	708 (348–1,562)	491 (233–1,119)	936 (457–1970)
Mortality	491,774 (124968–1,330,182)	163,343 (40664–466,660)	328,431 (83715–862,460)	35 (9–93)	22 (6–64)	47 (12–124)

**Figure 1:**
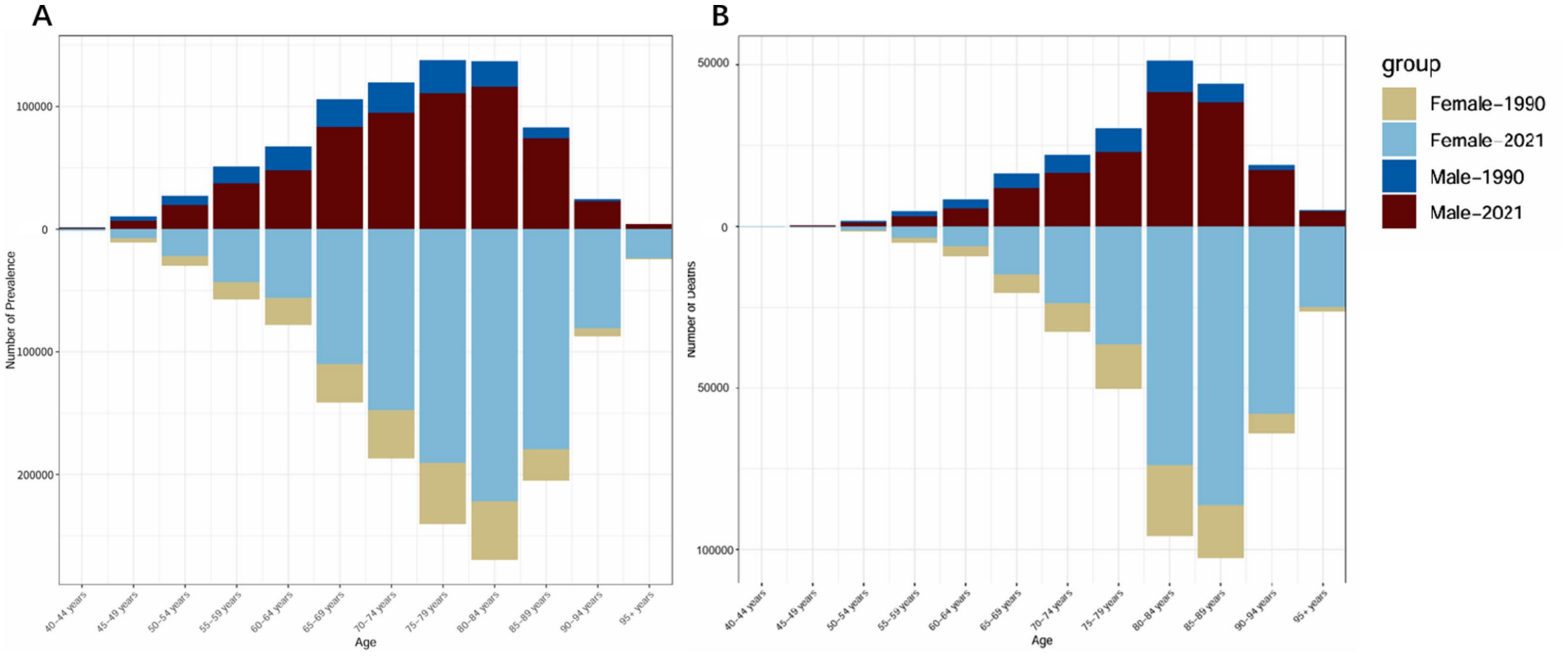
Comparison of the number of cases and deaths due to ADRD in China by gender and age group in 1990 and 2021. **(A)** Total number of cases. **(B)** Total number of deaths.

Figure 1 illustrates the number of ADRD cases and deaths across different age groups in 1990 and 2021. The data show that the number of ADRD cases rises rapidly from ages 40 to 80, peaking in the 65–85 age group, and gradually declines after the 80–84 age group. A similar trend is observed for deaths, with the majority concentrated in the 80–89 age group and declining after 80–84. The gender disparities in case and death numbers align with the findings in Table 1, where females consistently exhibit higher rates than males. Notably, compared to 1990, both male and female case and death numbers in 2021 demonstrated significant reductions.

### Joinpoint regression analysis

**Figure 2:**
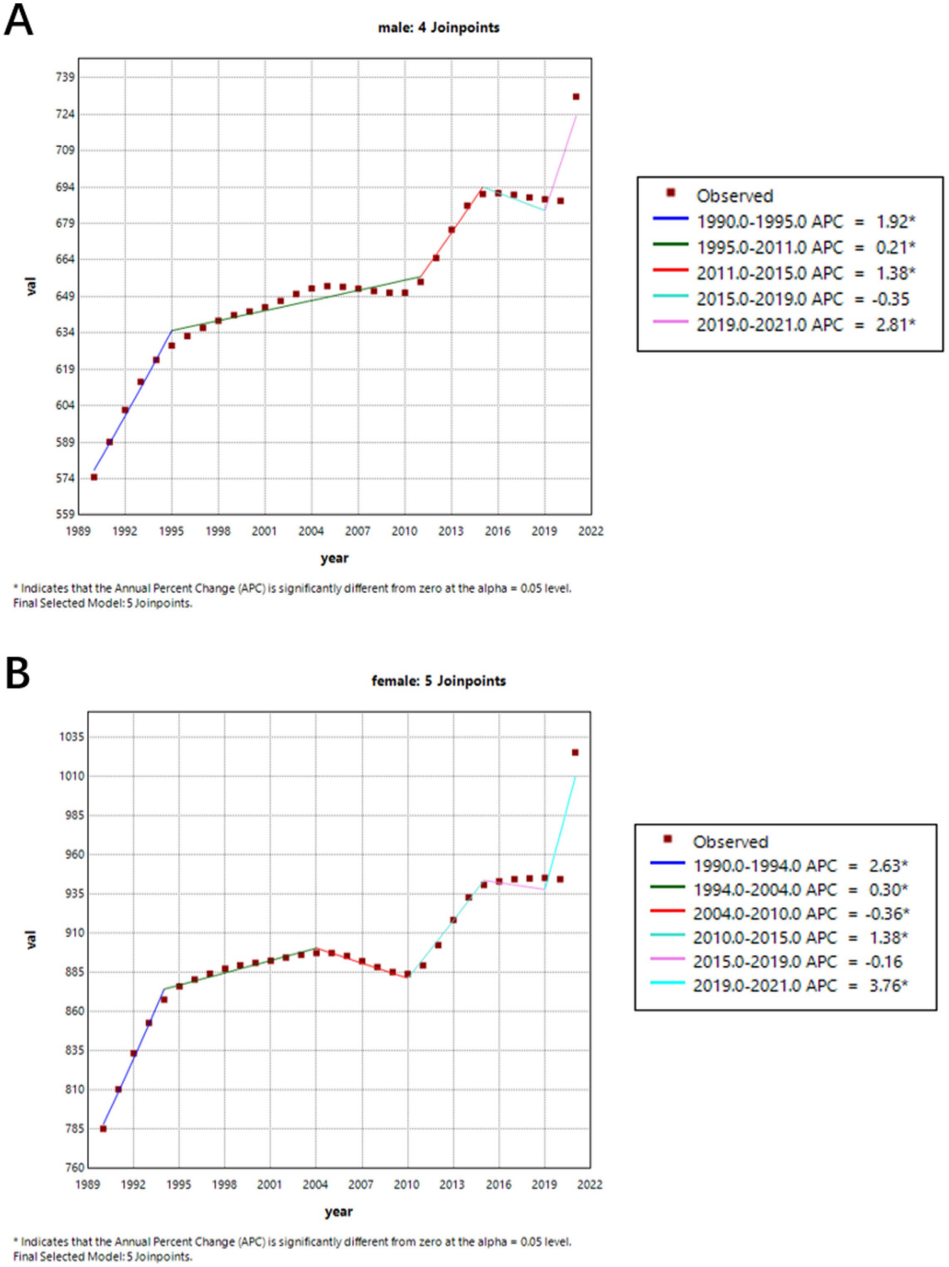
Gender-specific age-standardized prevalence rates of Alzheimer’s disease and other dementia (ADRD) in China from 1990 to 2021. **(A)** Female; **(B)** Male.

Figure 2 shows the trend of age-standardized prevalence from 1990 to 2021. We can observe that the prevalence of ADRD increased in both males (APC = 3.51%) and females (APC = 3.95%) from 1990 to 2015. However, from 2015 to 2019, both genders experienced a slight decline in prevalence (males APC = −0.35, 95% CI = −1.0378, 0.3427; females APC = −0.16, 95% CI = −0.4123, 0.1018), followed by a sharp increase between 2019 and 2021. Males experienced an APC of 2.81% (95% CI = 1.3776–4.2537) and females experienced an APC of 3.76% (95% CI = 3.2141–4.3168).

**Figure 3:**
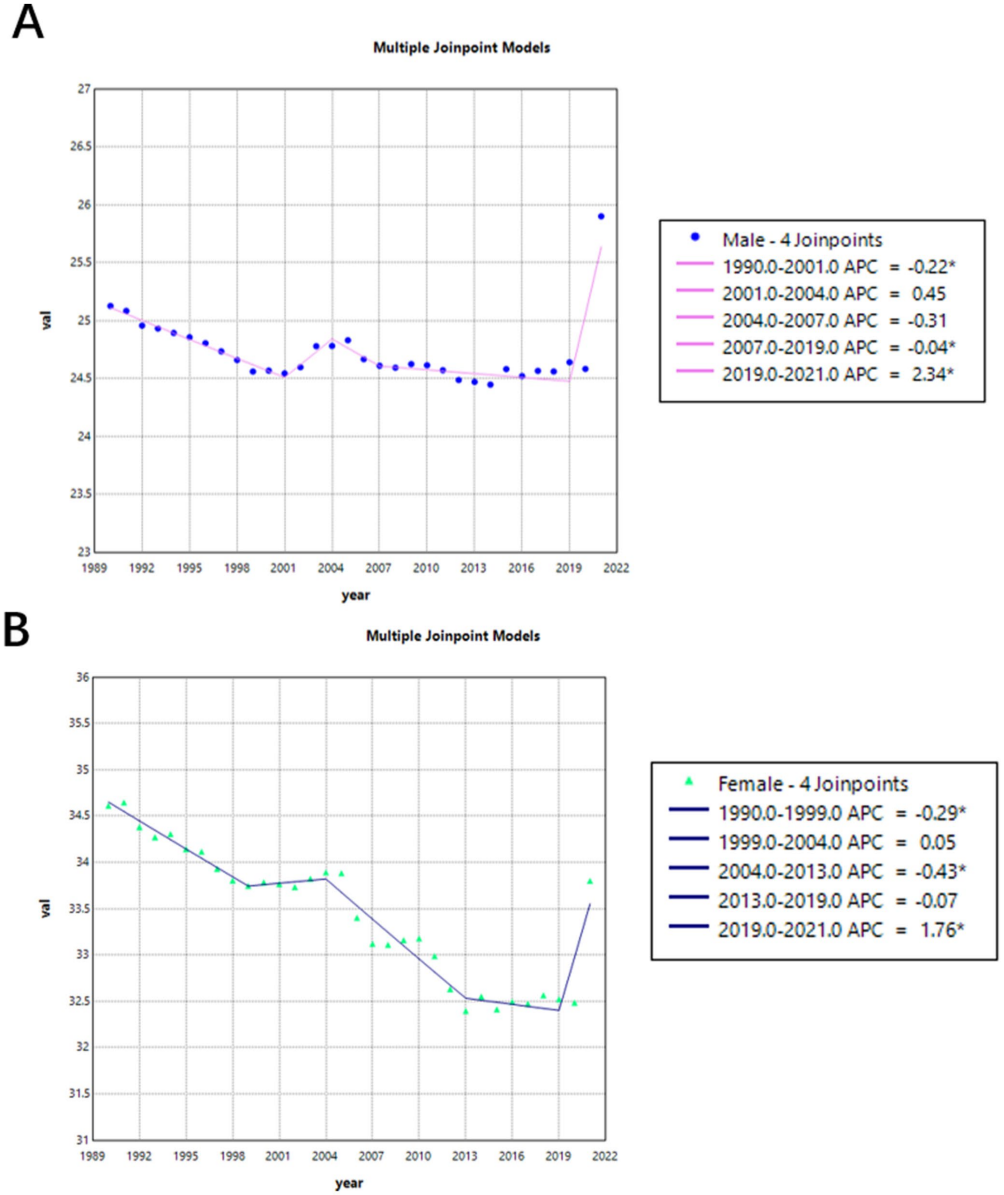
Gender-specific age-standardized mortality rates of Alzheimer’s disease and other dementia (ADRD) in China from 1990 to 2021. **(A)** Female; **(B)** Male.

Figure 3 shows the trend in age-standardized mortality rates from 1990 to 2021. Before 2019, mortality rates for both males (APC = −0.07%) and females (APC = −0.24%) were on the decline. But during 2001 and 2004, mortality rates for both female and male were increased, though this trend was not statistically significant. And between 2019 and 2021, there was a significant increase in mortality rates for both males (APC = 2.34, 95% CI = 1.7863–2.9032) and females (APC = 1.76, 95% CI = 0.6925–2.8423).

Table 2 shows specific changes of Figures 2, 3.

**Table 2:** Specific changes of Alzheimer’s disease and other dementia in China by age group from 1990 to 2021.

Sex	Age-standardized incidence rate (ASIR)		Age-standardized prevalence rate (ASPR)		Age-standardized mortality rate (ASMR)	
	Year	APC (95%CI)	Year	APC (95%CI)	Year	APC (95%CI)
Female	1990–1995	2.0374* (1.7203, 2.3555)	1990–2004	2.6305* (2.4702, 2.7911)	1990–2019	−0.2418* (−0.2668, -0.2169)
	1995–2011	0.0270 (−0.0327, 0.0867)	1994–2004	0.2951* (0.2494, 0.3409)	2019–2021	1.9698* (0.3958, 3.5685)
	2011–2015	1.3658* (0.6513, 2.0854)	2004–2010	−0.3588* (−0.4718, -0.2458)		
	2015–2019	−0.1207 (−0.8268, 0.5904)	2010–2015	1.3775* (1.2147, 1.5405)		
	2019–2021	3.2761* (1.8217, 4.7513)	2015–2019	−0.1556 (−0.4123, 0.1018)		
			2019–2021	3.7640* (3.2141, 4.3168)		
Male	1990–1994	1.9884* (1.8516, 2.1253)	1990–1994	2.1250* (1.9959, 2.2543)	1990–2019	−0.0700* (−0.0898, -0.0502)
	1994–2004	0.4016* (0.3617, 0.4415)	1994–2004	0.4238* (0.3867, 0.4610)	2019–2021	2.4605* (1.1932, 3.7437)
	2004–2010	−0.1374* (−0.2378, -0.0368)	2004–2010	−0.1111* (−0.2034, -0.0187)		
	2010–2015	1.4116* (1.2683, 1.5551)	2010–2015	1.3289* (1.1960, 1.4619)		
	2015–2019	−0.2801* (−0.5034, -0.0563)	2015–2019	−0.3270* (−0.5383, -0.1153)		
	2019–2021	2.5751* (2.1146, 3.0377)	2019–2021	2.7921* (2.3527, 3.2335)		
Total	1990–1995	1.8681* (1.5620, 2.1753)	1990–1994	2.4242* (2.2865, 2.5620)	1990–1999	−0.3146* (−0.397, -0.2318)
	1995–2011	0.0590* (0.0008, 0.1172)	1994–2005	0.2887* (0.2553, 0.3221)	1999–2004	0.1412 (−0.1478, 0.4310)
	2011–2015	1.3420* (0.6422, 2.0467)	2005–2010	−0.4125* (−0.5507, -0.2742)	2004–2013	−0.4389* (−0.5369, -0.3408)
	2015–2019	−0.1732 (−0.8625, 0.5210)	2010–2015	1.3182* (1.1767, 1.4600)	2013–2019	−0.0657 (−0.2631, 0.1321)
	2019–2021	2.9431* (1.5070, 4.3996)	2015–2019	−0.2083 (−0.4311, 0.0151)	2019–2021	1.9251* (1.0088, 2.8497)
			2019–2021	3.4229* (2.9551, 3.8929)		

^*^ *p* < 0.05.

Table 3 shows the AAPC for age-standardized incidence, prevalence, and mortality rates from 1990 to 2021. It is apparent that females have a higher prevalence rate than males, while their incidence and mortality rates are lower.

**Table 3:** Specific average annual percentage change (AAPC) of Alzheimer’s disease and other dementia in China by gender from 1990 to 2021.

Sex	Female	Male	Total
Incidence	0.7092* (0.5505, 0.8681)	0.7132* (0.6621, 0.7643)	0.6680* (0.5125, 0.8237)
Prevalence	0.8027* (0.7437, 0.8617)	0.7373* (0.6892, 0.7853)	0.7489* (0.6971, 0.8009)
Mortality	−0.1006* (−0.1989, -0.0022)	0.0913* (0.0126, 0.1702)	−0.0861* (−0.1716, -0.0005)

^*^ *p* < 0.05.

### The burden of ADRD in China compared to the global situation

**Figure 4:**
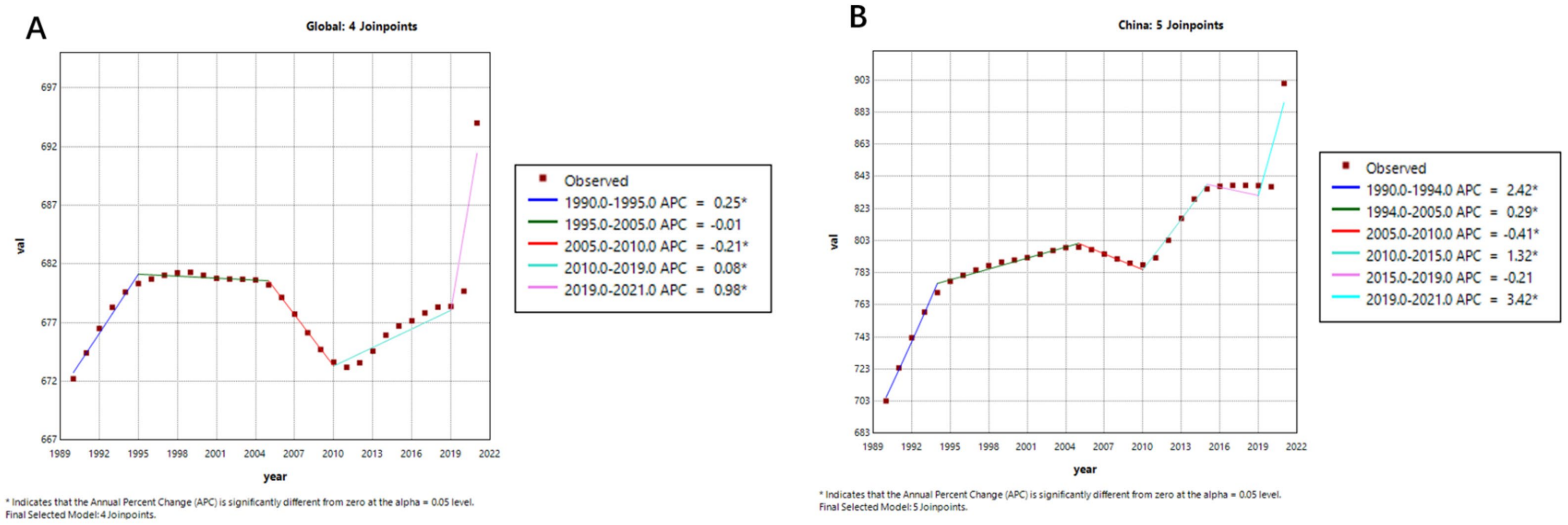
Age-standardized prevalence trends of Alzheimer’s disease and related dementias (ADRD) in China and globally. **(A)** Global; **(B)** China.

**Figure 5:**
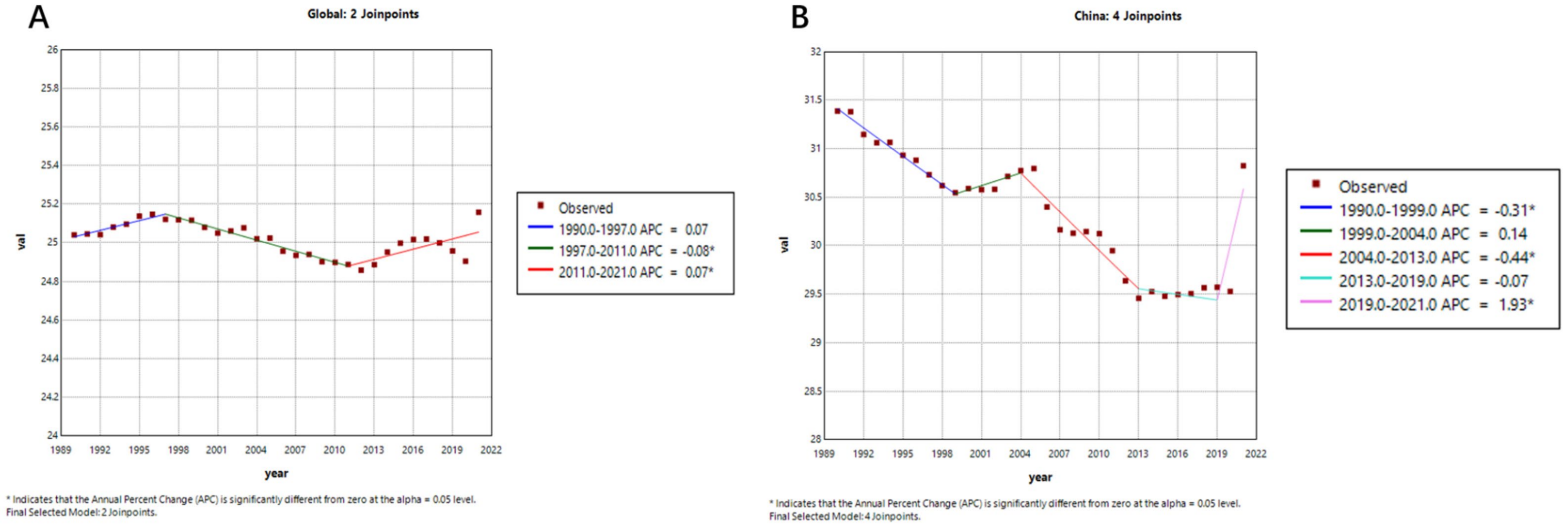
Age-standardized mortality trends of Alzheimer’s disease and related dementias (ADRD) in China and globally. **(A)** Global; **(B)** China.

Figures 4, 5 illustrate the comparative trends in age-standardized prevalence and mortality rates of Alzheimer’s disease and related dementias (ADRD) between China and the global population.

As shown in Figures 4A,B, after entering the 21st century, prior to the COVID-19 pandemic, the global age-standardized prevalence of ADRD displayed an overall declining trend. A more detailed segmented analysis reveals that during 2005–2010, both China (Figure 4B, APC = −0.41%) and the global average (Figure 4A, APC = −0.21%) experienced a slight decrease in prevalence. And after that, from 2010 to 2019, there was a significant upward trajectory—most notably in China, where the prevalence surged (APC = 4.53%), compared to a more modest global increase (APC = 1.06%). This indicates that the disease burden has grown more rapidly in China in recent years.

Figure 5 further highlights the contrasting trends in ADRD-related mortality. Globally (Figure 5A), the age-standardized mortality rate remained relatively stable from 1990 to 2019, with an APC of 0.0031% (95% CI: −0.0194 to 0.0257), suggesting little net change. In contrast, China’s mortality curve (Figure 5B) reveals a gradual but persistent downward trend, despite an APC value of −0.0861% (95% CI: −0.1716 to −0.0005), which may reflect the cumulative impact of an aging population and evolving diagnostic practices.

### Risk factors analysis

**Figure 6:**
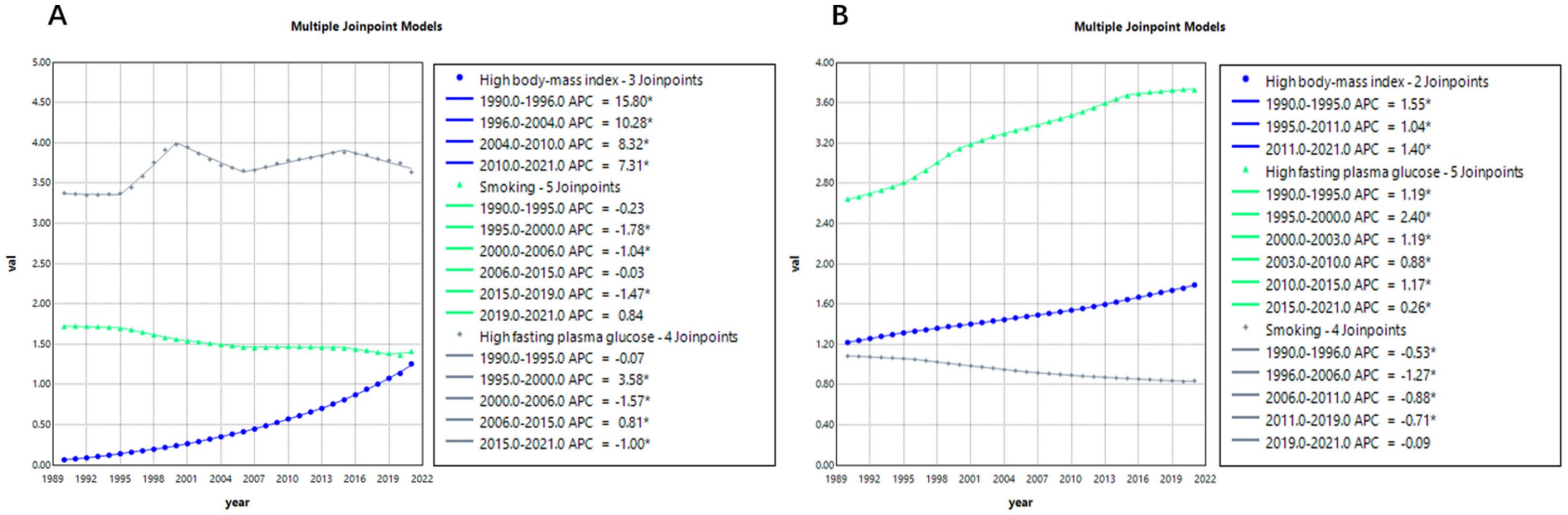
Analysis of risk factors for ADRD mortality. **(A)** China, **(B)** Global.

Figure 6 presents the joinpoint regression analysis results of age-standardized mortality rates (ASMRs) for Alzheimer’s disease and related dementias (ADRD) attributable to three risk factors: smoking, high BMI, and high fasting plasma glucose (FPG).

According to Figure 6A, between 1990 and 2021 in China, the average annual percentage changes (AAPC) in ADRD-related ASMRs due to smoking, high BMI, and high FPG were −0.6723% (95% CI: −0.7348, −0.6258), 9.8737% (95% CI: 9.7749, 10.0147), and 0.2887% (95% CI: 0.2437, 0.3332), respectively. Among the three, high BMI (obesity) contributed the greatest burden, with rates continuing to rise between 2004 and 2021, although the growth rate has been declining. The risk associated with smoking has steadily decreased throughout the observation period and remained relatively stable compared to the other two factors. In contrast, mortality risk due to high FPG fluctuated during 1990–2021—significantly increasing from 1995 to 2000 (APC = 3.58%), then slightly declining from 2000 to 2006 (APC = −1.57%), followed by more minor fluctuations.

Further comparing Figures 6A,B, the impacts of these three risk factors between China and the global average are observed. Globally, the AAPCs of ADRD-related ASMRs due to smoking, high BMI, and high FPG were −0.8431% (95% CI: −0.8701, −0.8161), 1.2405% (95% CI: 1.2221, 1.2588), and 1.1319% (95% CI: 1.0816, 1.1822), respectively. Regarding smoking, China and the global trend are generally aligned, but in recent years, China has shown signs of a rebound (APC = 0.84%), while globally, the risk continues to decline (APC = −0.71%). For high BMI, China’s growth rate far exceeds the global average. Meanwhile, globally, the risk associated with high FPG continues to grow steadily but with less volatility compared to China.

### Age-period-cohort analysis

**Figure 7:**
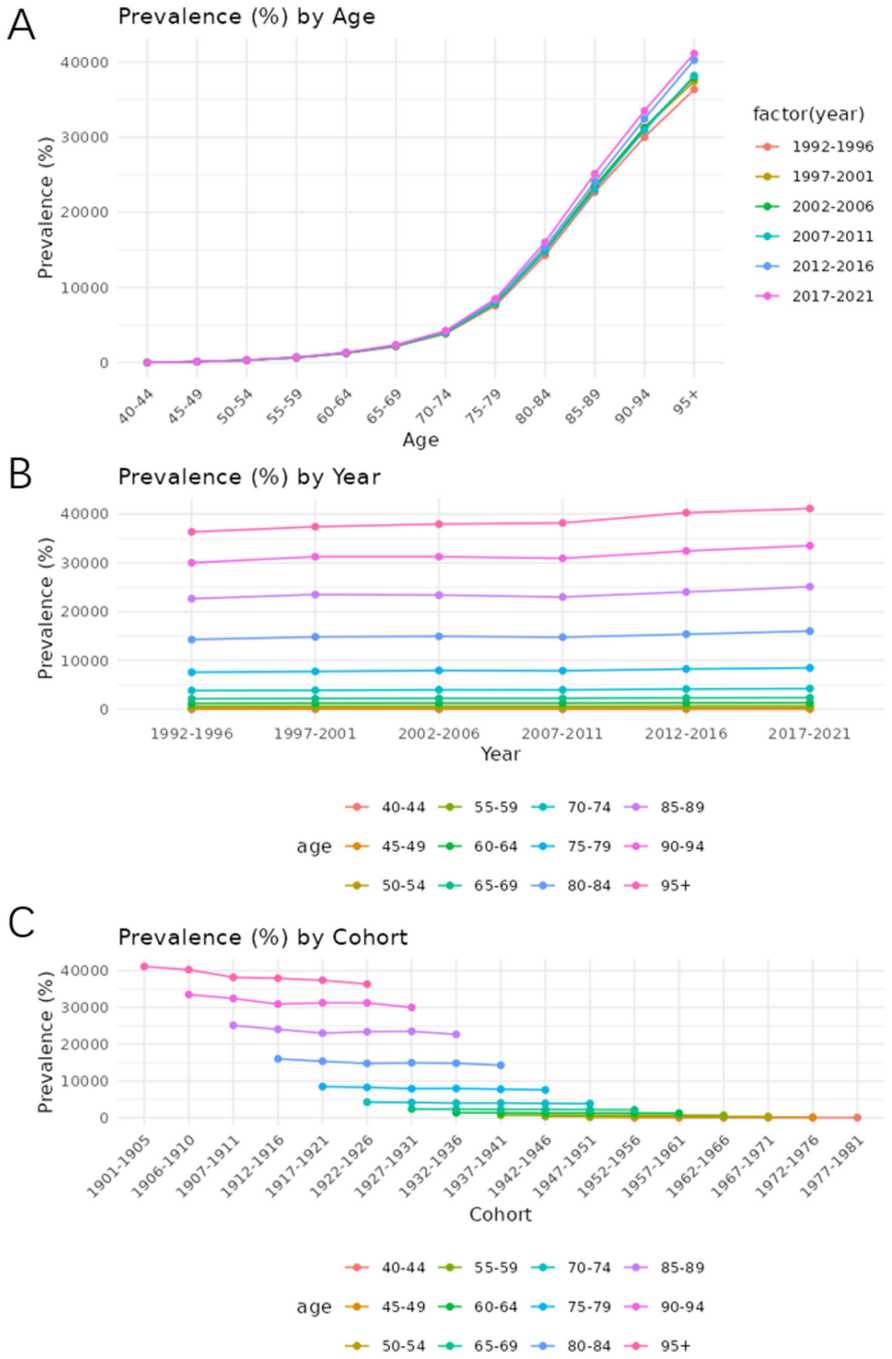
Age-period-cohort analysis of the prevalence rate of Alzheimer’s disease and other dementia in China. **(A)** Age, **(B)** Period, **(C)** Cohort.

**Figure 8:**
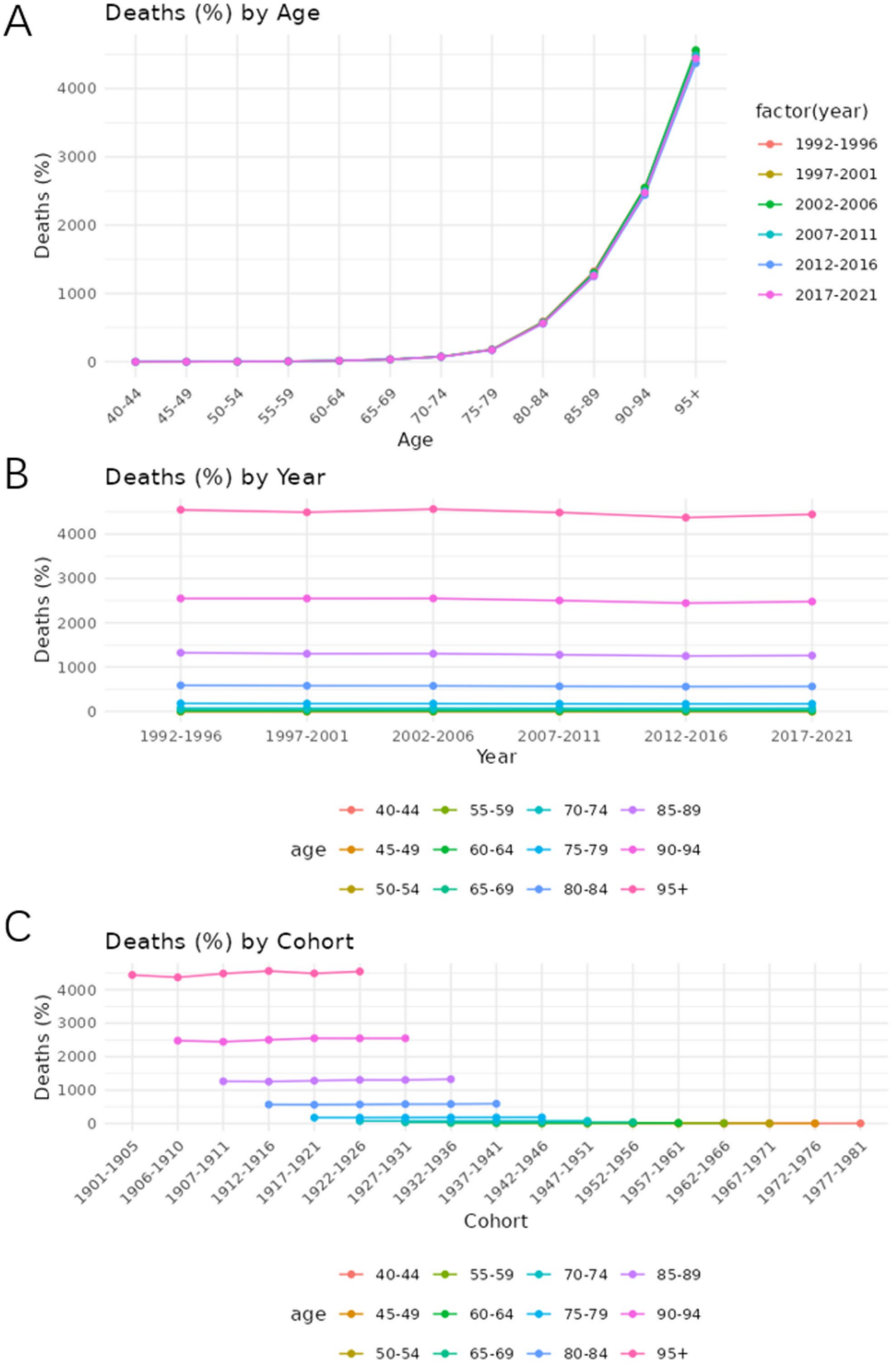
Age-period-cohort analysis of the mortality rate of Alzheimer’s disease and other dementia in China. **(A)** Age, **(B)** Period, **(C)** Cohort.

Figures 7, 8 show the trends in prevalence and mortality rates for different years, categorized by age, from 1990 to 2021. Figures 7A, 8A illustrated the trends of prevalence rate and mortality rate by age. In both figures, an upward trend is observed in prevalence and mortality rate. As we can see, the prevalence of Alzheimer’s disease and other dementias is steadily increasing. Individuals aged 85 and older experience the highest rates of both death and disease. For these age groups, mortality rates rise sharply, reflecting the greater vulnerability of older individuals to various health conditions. Similarly, disease prevalence follows a similar trajectory, with chronic conditions becoming more common as age increases.

Figures 7B, 8B show how prevalence and mortality rate change by year. In Figure 7B, a slight upward trend is observed, particularly in the most recent periods (2012–2021). The 85–89 age group stands out in both mortality and prevalence trends, with an observable increase in rates during this time frame.

Figures 7C, 8C describe the cohort trends of incidence and mortality rates for Alzheimer’s Disease and other dementia in different age groups. The cohort analysis indicates a significant difference in both mortality and disease prevalence between older and more recent cohorts. Those born before 1950 show much higher mortality rates and disease prevalence compared to those who born after 1960. This difference is most evident in the older age groups such as group 75–79 and 85–89 and can be attributed to the historical lack of access to advanced healthcare, poorer living conditions, and fewer preventive health measures available to earlier generations.

### 2022–2040 forecast of Alzheimer’s disease and other dementia prevalence and mortality trends in China

#### Prevalence forecast analysis

**Figure 9:**
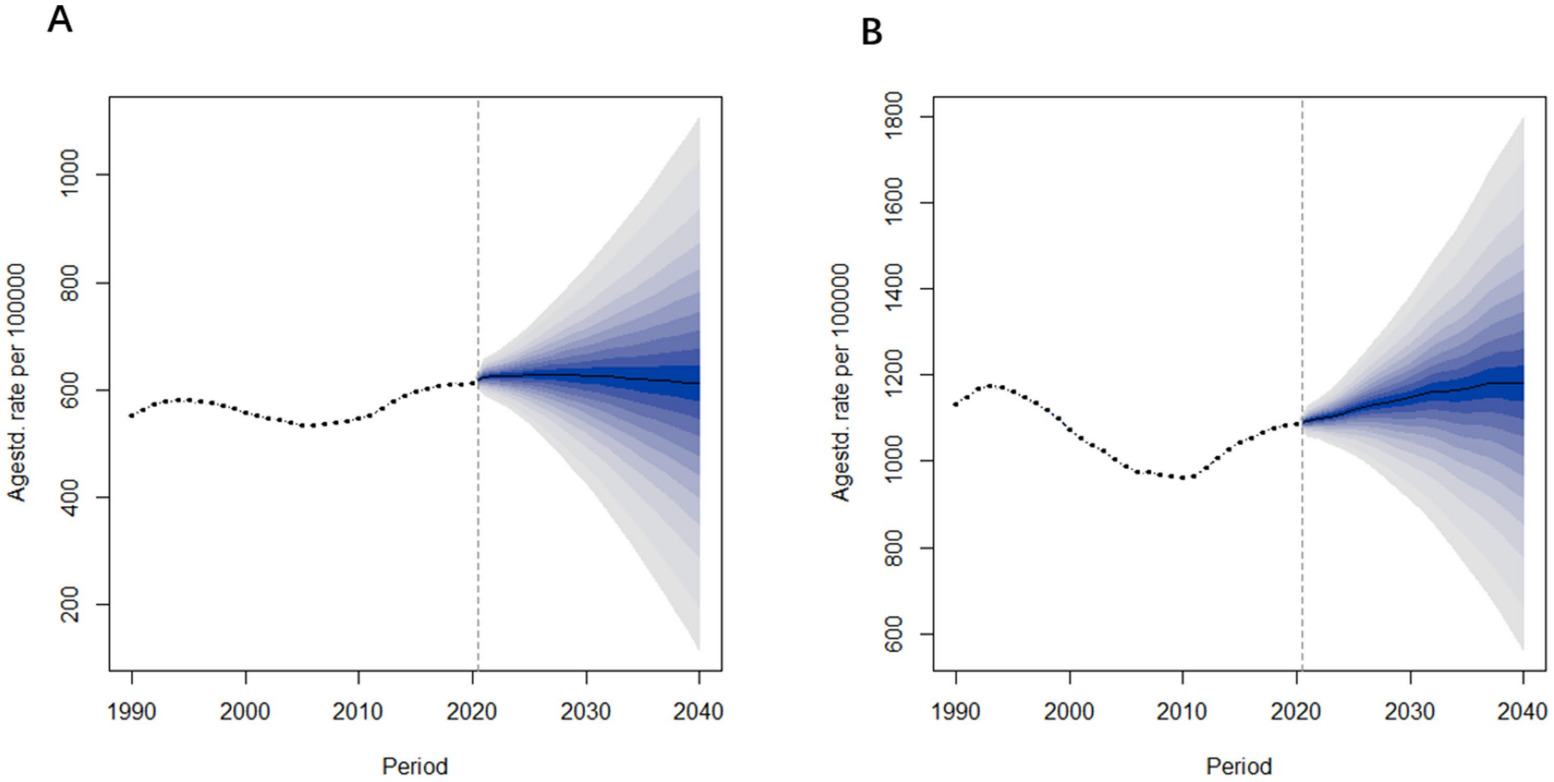
Age-standardized prevalence rate predictions of Alzheimer’s disease and other dementia in China from 2022 to 2040. **(A)** Male **(B)** Female.

According to the forecast results shown in Figure 9, the prevalence of Alzheimer’s Disease and other dementia in China is expected to increase over the next three to 5 years, followed by gender-specific variations. Specifically, the age-standardized prevalence rate for males is projected to decline after 2027, decreasing from 623.9 per 100,000 in 2021 to 611.1 per 100,000 in 2040. In contrast, the prevalence rate for females is expected to continue rising, with the increase slowing down as it approaches 2040. The age-standardized prevalence rate for females is predicted to rise from 1,094 per 100,000 in 2021 to 1,180 per 100,000 in 2040.

#### Mortality forecast analysis

**Figure 10:**
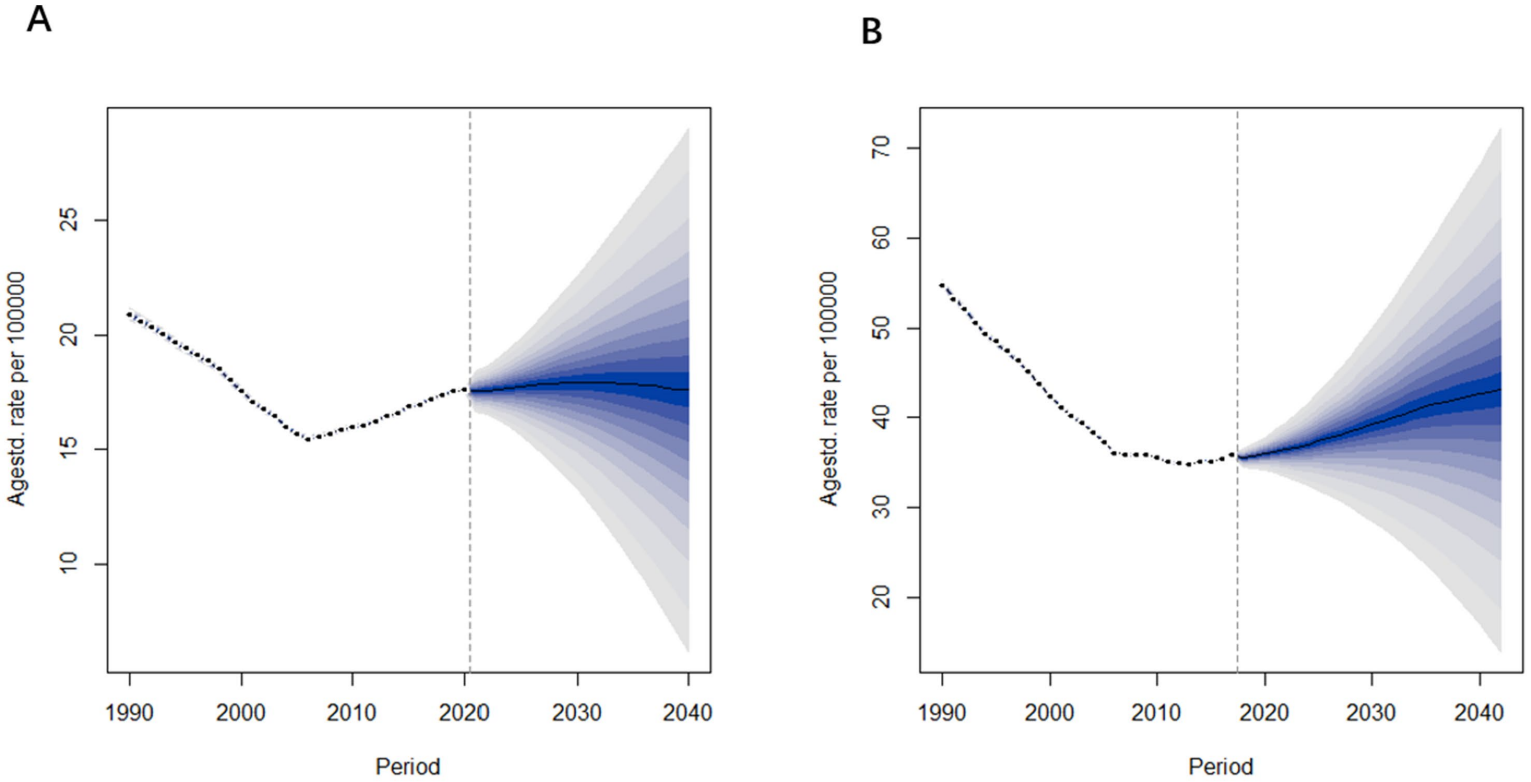
Age-standardized mortality rate predictions of Alzheimer’s disease and other dementia in China from 2022 to 2040. **(A)** Male **(B)** Female.

Figure 10 presents the projected age-standardized mortality rates for Alzheimer’s Disease and other dementia in China from 2022 to 2040. The trends in mortality rates are similar to those observed in prevalence rates. Specifically, the age-standardized mortality rate for males is projected to remain relatively stable, changing from 17.5 per 100,000 in 2021 to 17.6 per 100,000 in 2040. In contrast, the mortality rate for females is expected to increase, rising from 36.2 per 100,000 in 2021 to 43.1 per 100,000 in 2040.

From these projections, it is evident that both the prevalence and mortality rates among females are significantly higher than those of males, with female rates also showing a greater rate of increase over time. In contrast, the prevalence and mortality rates for males remain relatively stable.

## Discussion

This study reveals that between 1990 and 2021, the incidence and prevalence of Alzheimer’s disease (AD) and other dementia (ADRD) in China exhibited a general upward trend. These trends are broadly in line with global and regional findings, yet they also highlight some China-specific features. In comparison to global data, the increase in China has been particularly steep. As reported in previous studies, China’s ADRD burden grew threefold by 2021, outpacing the global doubling during the same period (6). However, from 2015 to 2019, a slight decline in both incidence and prevalence rates was observed, followed by a sharp increase after 2019. The most plausible explanation for this sudden shift is the impact of the COVID-19 pandemic, which placed an immense burden on healthcare resources, increased caregiving costs, and severely affected the quality of life of dementia patients (18).

Similarly, mortality rates showed an overall decline before 2019 but experienced a significant surge between 2019 and 2021. We observed that male ADRD mortality declined pre-2019. This is contrary to the conclusions drawn by previous, which found that crude mortality in men continued to rise between 2011 and 2020 (19). It suggests that any perceived declines in age-standardized rates have been small or non-significant.

Analysis using the Joinpoint regression method further supports that, excluding the extraordinary period of the pandemic, the long-term trajectory of incidence and prevalence continues to rise. This trend is largely driven by the accelerating aging of China’s population. As demographic shifts push China into a stage of deep aging, the number of individuals suffering from AD and other dementia continues to increase, positioning the country among those with the highest dementia burden worldwide (20). This demographic shift directly correlates with rising incidence and prevalence rates, yet China’s geriatric and rehabilitation medical services have not adequately adapted to meet these challenges, leading to significant shortages in healthcare resources (4, 20).

Interestingly, prior to COVID-19, mortality rates showed a downward trend, with a decrease in both total deaths and overall prevalence. This could be attributed to several factors, including increased health screenings at local and community levels, which may have improved early diagnosis rates. However, mortality statistics related to AD and other dementia may be underestimated, as deaths in older adult populations are often attributed to other conditions such as cardiovascular diseases and cancer. This aligns with previous studies suggesting that dementia-related deaths may be underreported (9). Moving forward, improvements in medical service coverage, public health awareness, and early screening programs could contribute to a continued gradual decline in mortality rates (21).

China’s burden of modifiable risk factors is distinct. Our analysis indicates that mortality linked to high fasting plasma glucose, high body mass index (BMI), and smoking is increasing—with obesity and diabetes emerging as the most significant drivers in recent years. As previous study specifically highlighted, high blood glucose is the dominant modifiable risk factor for dementia in China (6, 22). Perhaps it is because it directly affects brain health and accelerates neurodegenerative changes. Although smoking and a high BMI are also major risk factors, their effects are usually indirect and may vary at different stages of an individual’s life cycle (23). Furthermore, different studies may focus on different datasets or time Windows of risk factors, which may affect the differences in conclusions. Therefore, it is recommended that multiple metabolic factors be combined in future research and their combined effects be considered to assess the risk factors of dementia more comprehensively. At the same time, smoking as a leading risk factor, particularly for men, is also recognized worldwide as the main risk factor for men (24). These comparisons support our risk factor findings.

Currently, China is characterized by “high growth rate and strong volatility” in mortality risks linked to obesity and high blood glucose, while the risk from smoking is facing a rebound trend. In contrast, global trends are more gradual, though common challenges remain. Going forward, China must tailor chronic disease prevention strategies to national conditions while also drawing on international experiences to ensure the long-term, stable control of these risk factors. Although the growth rate of obesity-related mortality has decelerated since 2004, the continued upward trajectory suggests that obesity remains a major and growing contributor to ADRD burden in China. This reflects broader shifts in lifestyle and diet patterns associated with rapid urbanization and economic development, consistent with previous findings on metabolic risk factor trends in China.

Age-period-cohort (APC) analysis indicates that while prevalence rates have increased across different age groups over time, the growth in mortality significantly outpaced the rise in prevalence. This suggests that the number of people dying from dementia is increasing at a faster rate than those living with it, leading to a relative decline in dementia-related prevalence rates. The statistical analysis further confirms that in China, both prevalence and mortality rates increase significantly with age, especially among those aged 85–89. In some articles that reached the same conclusion as me, they believed that this pattern is likely due to the accumulation of age-related risk factors such as cerebrovascular disease, hypertension, and obesity, which significantly contribute to dementia onset and progression (25). Additionally, older adults are more susceptible to depression, sleep disorders, and other factors that can accelerate cognitive decline (26).

According to Bayesian Age-Period-Cohort (BAPC) model predictions, the prevalence and mortality of AD and other dementia in China will continue to rise between 2021 and 2040, with a more pronounced increase among women compared to men. Apart from the overarching aging trend, socioeconomic status and lifestyle factors also play critical roles in dementia risk (27, 28). Smoking, alcohol consumption, poor dietary habits, and lack of physical activity have all been linked to increased AD risk, particularly when unhealthy habits persist from a young age (27). As China continues to develop economically, efforts should be made to minimize social disparities and encourage healthier lifestyles. Raising public awareness and promoting healthy behaviors—such as reducing excessive screen time, engaging in social activities, and maintaining cognitive stimulation—can help mitigate future dementia risks (29). Government initiatives should also incorporate these insights into public education, social policies, and healthcare reforms.

The findings indicate significant gender disparities in dementia incidence, prevalence, and mortality. Across all metrics, women consistently exhibit higher rates than men, with greater fluctuations over time, as same as previous studies (30). Projections suggest that the disease burden among women will continue to rise, whereas it is expected to stabilize among men. These observations align with previous research suggesting biological and hormonal differences between men and women contribute to the disparity (9, 31). One potential explanation involves the role of estrogen. During perimenopause, estrogen levels become highly variable, disrupting metabolic and inflammatory pathways linked to cognitive function. Postmenopausal estrogen depletion, coupled with elevated luteinizing hormone and follicle-stimulating hormone levels, may further exacerbate cognitive decline (32, 33). Given these findings, there is an urgent need for more targeted research into female brain health, particularly concerning the prevention of AD and other dementia in postmenopausal women (33).

In 2017, the World Health Organization (WHO) launched the Global Action Plan on Dementia (GAPD), calling for countries to develop or update national dementia strategies by 2025 (34). However, the implementation of such policies varies widely based on national income levels and socio-economic disparities. Factors such as race, occupation, and family environment can influence dementia prevalence, making it challenging to establish a one-size-fits-all approach (34–37).

Over the past two decades, the incidence of dementia among the older adult has declined in many developed Western countries, attributed to improved public health measures, education, and lifestyle changes (38). European nations, in particular, have implemented strategic frameworks emphasizing treatment, education, and research, which have helped reduce dementia rates. These measures have contributed to increased awareness and preventive strategies that have positively impacted long-term outcomes (38, 39). However, low- and middle-income countries actually account for a larger proportion of the dementia population, yet lack adequate policies for the prevention, treatment, and care of Alzheimer’s disease and related dementia (ADRD) (40). China—the world’s largest developing country—has also made strides in addressing AD and other dementia, implementing policies aimed at long-term care, healthcare resource allocation, and older adult health services (20). Investments in chronic disease prevention and education have increased, leading to higher average educational attainment and reduced educational inequality. These factors play a role in alleviating the burden of AD and related dementia (31). Further collaboration between health economists and clinical experts is essential to refine public health insurance plans, optimize care provisions, and reduce patient burdens, ensuring equitable access to necessary services (41).

### Implications for national public health planning and future research

Early diagnosis and intervention remain key in managing AD and other dementia. Strengthening screening programs, enhancing rehabilitation care, and improving patient quality of life can help alleviate disease burdens and potentially reduce mortality rates. China should integrate dementia into public health policy, emphasizing early detection and management of cognitive impairment among seniors. And moreover, the healthcare system must prepare for rising long-term care needs: most dementia care in China is currently provided by families, leading to substantial caregiver burden (42).

Future research should build on this work by incorporating more recent data and granular analyses. Longitudinal cohort studies in China should continue to monitor incidence and risk factors, ideally including biomarkers (43, 44). Additionally, establishing national dementia registries would help track cases more accurately over time. As previous study noted, China faces both challenges and opportunities in building such surveillance systems, including data standardization and integrating registries into public health infrastructure (45). Finally, research into culturally tailored prevention like traditional medicine effects and cost-effective care models will support evidence-based policymaking.

This study has several limitations. First, it does not distinguish between Alzheimer’s disease and other forms of dementia, leading to a generalized analysis of dementia burden rather than a condition-specific breakdown. Second, in both the current analysis and future projections, the impact of relevant influencing factors on trends was not considered. Moreover, due to the lack of risk factor data related to prevalence in GBD, only mortality rates could be analyzed. Additionally, China’s vast geographic and ethnic diversity means that regional and ethnic variations could not be adequately analyzed. Lastly, due to limited sample sizes for certain age groups (40–44 and 95+), some findings may be subject to bias.

## Conclusion

This study analyzes China’s Alzheimer’s Disease and other dementia burden using data from GBD 2021 and forecasts future trends. The results indicate that the disease burden of AD and related dementia will continue to grow. Effective intervention strategies are urgently needed to address the challenges posed by an aging population. The BAPC model predicts significant gender differences in the disease burden trends by 2040, with more stable changes in males. Thus, tailored strategies targeting gender differences are necessary. This study provides valuable insights for the prevention, treatment, and care of AD and other dementia in China.

## Data Availability

Publicly available datasets were analyzed in this study. This data can be found at: GBD2021 https://vizhub.healthdata.org/gbd-results/.

## References

[1] Soria Lopez JA González HM Léger GC. Alzheimer's disease. Handb Clin Neurol. (2019). 167:231–55. doi: 10.1016/B978-0-12-804766-8.00013-3 31753135

[2] Weller J Budson A. Current understanding of Alzheimer’s disease diagnosis and treatment. F1000Res. (2018) 7:F1000 Faculty Rev-1161. doi: 10.12688/f1000research.14506.1, PMID: 30135715 PMC6073093

[3] Li X. Global, regional, and national burden of Alzheimer's disease and other dementias, 1990–2019. Front Aging Neurosci. (2022) 14:937486. doi: 10.3389/fnagi.2022.937486 PMC9588915 36299608

[4] Chen X Giles J Yao Y Yip W Meng Q Berkman L. The path to healthy ageing in China: a Peking University–lancet commission. Lancet. (2022) 400:1967–2006. doi: 10.1016/S0140-6736(22)01546-X, PMID: 36423650 PMC9801271

[5] Wang Gang WG Cheng Qi CQ Zhang Shi ZS Bai Li BL Zeng Jie ZJ Cui PeiJing CP. Economic impact of dementia in developing countries: an evaluation of Alzheimer-type dementia in Shanghai, China. J Alzheimers Dis. (2008) 15:109–15. doi: 10.3233/jad-2008-15109 18780971

[6] Liu S Geng D. A systematic analysis for disease burden, risk factors, and trend projection of Alzheimer's disease and other dementias in China and globally. PLoS One. (2025) 20:e0322574. doi: 10.1371/journal.pone.0322574, PMID: 40333703 PMC12057861

[7] Cao Y Yu F Lyu Y Lu X. Promising candidates from drug clinical trials: implications for clinical treatment of Alzheimer's disease in China. Front Neurol. (2022) 13:1034243. doi: 10.3389/fneur.2022.1034243 PMC9706102 36457865

[8] Vos T Lim SS Abbafati C Abbas KM Abbasi M Abbasifard M. Global burden of 369 diseases and injuries in 204 countries and territories, 1990–2019: a systematic analysis for the global burden of disease study 2019. Lancet. (2020) 396:1204–22. doi: 10.1016/S0140-6736(20)30925-9, PMID: 33069326 PMC7567026

[9] Bai RH Dong WY. Trends in mortality rates for Alzheimer's disease and other dementias over 30 years in China. Am J Alzheimers Dis Other Dement. (2021) 36. doi: 10.1177/15333175211044884, PMID: 34565197 PMC10581134

[10] Nichols E Steinmetz JD Vollset SE Fukutaki K Chalek J Abd-Allah F. Estimation of the global prevalence of dementia in 2019 and forecasted prevalence in 2050: an analysis for the global burden of disease study 2019. Lancet Public Health. (2022) 7:e105–25. doi: 10.1016/S2468-2667(21)00249-8, PMID: 34998485 PMC8810394

[11] Zhu Me. A GBD 2021 study of Alzheimer’s disease and other dementias attributable to metabolic risk factors and forecasts to 2045 in China. Front Public Health. (2025) 13:1575906. doi: 10.3389/fpubh.2025.1575906 PMC11998917 40236322

[12] Zhang Y Liu J Han X Jiang H Zhang L Hu J. Long-term trends in the burden of inflammatory bowel disease in China over three decades: a joinpoint regression and age-period-cohort analysis based on GBD 2019. Front Public Health. (2022) 10:994619. doi: 10.3389/fpubh.2022.994619, PMID: 36159285 PMC9490087

[13] Zhao Y Zhuang Z Yang L He D. Age-period-cohort analysis and projection of cancer mortality in Hong Kong, 1998–2030. BMJ Open. (2023) 13:e072751. doi: 10.1136/bmjopen-2023-072751, PMID: 37821140 PMC10583025

[14] Dhamnetiya D Patel P Jha RP Shri N Singh M Bhattacharyya K. Trends in incidence and mortality of tuberculosis in India over past three decades: a joinpoint and age-period-cohort analysis. BMC Pulm Med. (2021) 21:375. doi: 10.1186/s12890-021-01740-y, PMID: 34784911 PMC8597252

[15] Carstensen B. Age–period–cohort models for the Lexis diagram. Stat Med. (2007) 26:3018–45. doi: 10.1002/sim.2764, PMID: 17177166

[16] Pauw RD Lakha F Fletcher E Stockton DL Baird E Connolly S. Historic trends and future projections of the prevalence of adult excess weight in Scotland, 2003 to 2040: a Bayesian age-period-cohort modelling study. medRxiv. (2025):2025.01.07.24319409 10.1016/j.puhe.2025.105981 41061628

[17] Zhao X Lu X Ke L Meng X Ren L Connolly S. Analysis of the burden of hypertension in the elderly population globally and in China from 1990 to 2021 and prediction of future trends. J Peking Union Med College. (1990):1–17.

[18] Yang K Yang X Yin P Zhou M Tang Y. Temporal trend and attributable risk factors of Alzheimer's disease and other dementias burden in China: findings from the global burden of disease study 2021. Alzheimers Dement. (2024) 20:7871–84. doi: 10.1002/alz.14254, PMID: 39312279 PMC11567818

[19] Lv B Liang L Chen A Yang H Zhang X Guo F. Mortality of Alzheimer’s disease and other dementias in China: past and future decades. Int J Public Health. (2023) 68:1605129. doi: 10.3389/ijph.2023.1605129, PMID: 36816830 PMC9935610

[20] Wang G. China Alzheimer's Disease Report 2024. Diag Theory Prac. (2024) 23:219–56. doi: 10.16150/j.1671-2870.2024.03.001

[21] Lingxiu C Renyu L. Analysis and change trend prediction of laryngeal cancer disease burden in China from 1990 to 2021. J Math Med. (2025) 38:81–9.

[22] Guo J Wang P Gong J Sun W Han X Xu C. The disease burden, risk factors and future predictions of Alzheimer's disease and other types of dementia in Asia from 1990 to 2021. J Prev Alzheimers Dis. (2025) 12:100122. doi: 10.1016/j.tjpad.2025.100122, PMID: 40057462 PMC12183997

[23] 2020 Alzheimer's disease facts and figures. Alzheimers & Dement. (2020) 16:391–460. doi: 10.1002/alz.12068 32157811

[24] Zhong G Wang Y Zhang Y Guo JJ Zhao Y. Smoking Is Associated with an Increased Risk of Dementia: A Meta-Analysis of Prospective Cohort Studies with Investigation of Potential Effect Modifiers. PLoS One. (2015) 10:e0126169. doi: 10.1371/journal.pone.0126169 25763939 PMC4357455

[25] Qiu C Kivipelto M Von Strauss E. Epidemiology of Alzheimer's disease: occurrence, determinants, and strategies toward intervention. Dialogues Clin Neurosci. (2009) 11:111–28. doi: 10.31887/DCNS.2009.11.2/cqiu, PMID: 19585947 PMC3181909

[26] Alexopoulos GS. Depression in the elderly. Lancet. (2005) 365:1961–70. doi: 10.1016/S0140-6736(05)66665-2, PMID: 15936426

[27] Filippini T Vinceti M. Social disparities and unhealthy lifestyles increase risk of dementia, particularly at a young age. Lancet Healthy Longev. (2023) 4:E660–1. doi: 10.1016/S2666-7568(23)00233-7, PMID: 38042158

[28] Li R Li R Xie J Chen J Liu S Pan A. Associations of socioeconomic status and healthy lifestyle with incident early-onset and late-onset dementia: a prospective cohort study. Lancet Healthy Longevity. (2023) 4:e693–702. doi: 10.1016/S2666-7568(23)00211-8, PMID: 38042162

[29] Manwell LA Tadros M Ciccarelli TM Eikelboom R. Digital dementia in the internet generation: excessive screen time during brain development will increase the risk of Alzheimer's disease and related dementias in adulthood. J Integr Neurosci. (2022) 21:28. doi: 10.31083/j.jin2101028, PMID: 35164464

[30] Huque H Eramudugolla R Chidiac B Ee N Ehrenfeld L Matthews FE. Could country-level factors explain sex differences in dementia incidence and prevalence? A systematic review and Meta-analysis. J Alzheimers Dis. (2023) 91:1231–41. doi: 10.3233/JAD-220724, PMID: 36565114 PMC9986694

[31] Zhu ZY Zhu Z Zheng Z Zhou C Cao L Zhao G. Trends in prevalence and disability-adjusted life-years of Alzheimer's disease and other dementias in China from 1990 to 2019. Neuroepidemiology. (2023) 57:206–17. doi: 10.1159/000530593, PMID: 37231950

[32] Lopez-Lee C Torres ERS Carling G Gan L. Mechanisms of sex differences in Alzheimer’s disease. Neuron. (2024) 112:1208–21. doi: 10.1016/j.neuron.2024.01.024, PMID: 38402606 PMC11076015

[33] Barth C Crestol A de Lange A-MG Galea LAM. Sex steroids and the female brain across the lifespan: insights into risk of depression and Alzheimer's disease. Lancet Diabetes Endocrinol. (2023) 11:926–41. doi: 10.1016/S2213-8587(23)00224-3, PMID: 37865102

[34] Cahill S. WHO's global action plan on the public health response to dementia: some challenges and opportunities. Aging Ment Health. (2020) 24:197–9. doi: 10.1080/13607863.2018.1544213, PMID: 30600688

[35] Nair R Haynes VS Siadaty M Patel NC Fleisher AS Van Amerongen D. Retrospective assessment of patient characteristics and healthcare costs prior to a diagnosis of Alzheimer's disease in an administrative claims database. BMC Geriatr. (2018) 1–12. doi: 10.1186/s12877-018-0920-2 PMC6192320 30326851

[36] Keohane LM Nikpay S Braun K Cheng A Stevenson D Buntin MB. Association of Race and Income with incident diagnosis of Alzheimer's disease and related dementias among black and white older adults. J Appl Gerontol. (2023) 42:898–908. doi: 10.1177/07334648221142851, PMID: 36469682 PMC10081951

[37] Yaffe K Falvey C Harris TB Newman A Satterfield S Koster A. Effect of socioeconomic disparities on incidence of dementia among biracial older adults: prospective study. BMJ. (2013) 347. doi: 10.1136/bmj.f7051, PMID: 24355614 PMC3898154

[38] Contador I Buch-Vicente B del Ser T Llamas-Velasco S Villarejo-Galende A Benito-León J. Charting Alzheimer’s disease and dementia: epidemiological insights, risk factors and prevention pathways. J Clin Med. (2024) 13:4100. doi: 10.3390/jcm13144100, PMID: 39064140 PMC11278014

[39] Chirico I Chattat R Dostálová V Povolná P Holmerová I de Vugt ME. The integration of psychosocial care into National Dementia Strategies across Europe: evidence from the skills in DEmentia care (SiDECar) project. Int J Environ Res Public Health. (2021) 18:3422. doi: 10.3390/ijerph18073422, PMID: 33806158 PMC8036745

[40] Kalaria R Maestre G Mahinrad S Acosta DM Akinyemi RO Alladi S. The 2022 symposium on dementia and brain aging in low- and middle-income countries: highlights on research, diagnosis, care, and impact. Alzheimers Dement. (2024) 20:4290–314. doi: 10.1002/alz.13836, PMID: 38696263 PMC11180946

[41] Baicker K Simon K. Developing evidence-based health policy for dementia care. JAMA Health Forum. (2024) 5:e245252–2. doi: 10.1001/jamahealthforum.2024.5252, PMID: 39666359

[42] Xing B Li H Hua H Jiang R. Economic burden and quality of life of patients with dementia in China: a systematic review and meta-analysis. BMC Geriatr. (2024) 24:789. doi: 10.1186/s12877-024-05359-6, PMID: 39342118 PMC11437821

[43] Ye B Xu Y Chan WK Zhang Z Lobanov-Rostovsky S Curry N. Why are people with dementia overlooked in long-term care insurance policy in Guangzhou, China? BMC Health Serv Res. (2024) 24:1646. doi: 10.1186/s12913-024-12126-1, PMID: 39716249 PMC11668000

[44] Jia J Ning Y Chen M Wang S Yang H Li F. Biomarker changes during 20 years preceding Alzheimer’s disease. N Engl J Med. (2024) 390:712–22. doi: 10.1056/NEJMoa2310168, PMID: 38381674

[45] Ren R Qi J Lin S Liu X Yin P Wang Z. The China Alzheimer report 2022. Gen Psychiatr. (2022) 35:e100751. doi: 10.1136/gpsych-2022-100751, PMID: 35372787 PMC8919463

